# Tracing Recombinant Bovine Somatotropin Ab(Use) Through Gene Expression in Blood, Hair Follicles, and Milk Somatic Cells: A Matrix Comparison

**DOI:** 10.3390/molecules23071708

**Published:** 2018-07-13

**Authors:** Alexandre Lamas, Patricia Regal, Beatriz Vazquez, José Manuel Miranda, Alberto Cepeda, Carlos Manuel Franco

**Affiliations:** Laboratorio de Higiene, Inspección y Control de Alimentos, Dpto. de Química Analítica, Nutrición y Bromatología, Universidad de Santiago de Compostela, 27002 Lugo, Spain; beatriz.vazquez@usc.es (B.V.); josemanuel.miranda@usc.es (J.M.M.); alberto.cepeda@usc.es (A.C.); carlos.franco@usc.es (C.M.F.)

**Keywords:** rbST, nanoliter PCR, gene expression, screening, blood, hair, somatic cells

## Abstract

The use of recombinant bovine somatotropin (rbST) in dairy cattle is forbidden in the European Union. Due to the very low circulating concentration of rbST in treated animals, its direct detection is still a challenge. Therefore, the use of indirect methods to detect the ab(use) of rbST in dairy cattle appears as a good alternative. In the past few years, gene expression demonstrated its utility in screening the use of illicit substances in both humans and animals. In this study, a comparison of three types of matrices (milk somatic cells, blood, and hair follicles) was carried out to evaluate their potential use for routine control of rbST using 15 gene-expression profiles. A total of six rbST-treated cows and three control cows were included in the study. A subcutaneous injection containing 500 mg of rbST was administered to the treated group. Samples of the three matrices were collected before rbST administration, and at three and nine days after treatment. The quality of RNA extracted was higher in the blood and hair-follicle samples than in the milk somatic cells. In the three matrices, there were significant differences in the expression of some genes, with milk somatic cells and blood presenting the the best matrices. On this note, the cyclin D1 (*CCND1*), interleukin 1 beta (*IL-1β*), tumor necrosis factor (*TNF*), and insulin-like growth factor 1 receptor (*IGF-1R*) genes showed potential as biomarkers of rbST treatment. Therefore, blood, somatic cells, and follicle hair should be considered as promising sources of RNA, and can be used in gene-expression assays to routinely control the illicit use of rbST.

## 1. Introduction

The growth hormone (GH) is a single-chain polypeptide secreted by the anterior pituitary gland of all vertebrates. This hormone is involved in a wide range of biological activities, such as growth, energy metabolism, sexual maturation, and immunity. These actions are initiated upon the binding of GH to membrane-bound receptors located in various tissues, including liver, muscle, bone, or mammary tissue [1]. The GH-mediated actions in mammary tissue were studied in dairy cattle for many years. Furthermore, studies carried out in Russia and England at the beginning of the 20th century found that pituitary extracts from cows increased milk yield in dairy cattle [2,3]. This effect was due to the presence of growth hormone in the extract. In the 1980s, recombinant DNA technology allowed the production of large quantities of recombinant bovine somatotropin (rbST) in a cost-effective and efficient manner. The galactopoietic effect of the recombinant variants was demonstrated, and the routine use of rbST in dairy farms to increase the global milk yield started being evaluated [4]. Thus, in the year 1993, the Food and Drug Administration (FDA) approved the use of rbST in the United States.

Despite its capacity to enhance milk production in dairy farms, the use of rbST was controversial since the beginning. Some studies observed that the use of rbST could increase the incidence of mastitis and lameness in dairy farms, with a consequent deterioration of animal welfare [5]. Moreover, it was suggested that the increased presence of insulin-like growth factor 1 (IGF-1) in milk as a consequence of rbST use could have a detrimental effect in human health [6]. Therefore, while the use of rbST is allowed in various countries such as United States, Brazil, and Mexico, its use is banned in others such as Canada, Australia, and New Zealand. In 1999, the European Union (EU) definitively banned the use of rbST in Europe, invoking animal welfare reasons and its impact on European milk policy and consumer fears [7].

The prohibition of rbST use in the EU cannot prevent the illicit use of this substance in dairy farms. Consequently, the illegal use of rbST in dairy farms was detected in 2013 in Spain. The persons involved in this scandal introduced commercial injections from Mexico, where the use of this substance is allowed. Therefore, it is clear that rbST should be included in the European residue-control plans [8]. Analytical chemistry techniques combining chromatography and mass spectrometry are commonly used to detect the illicit use of substances in cattle (e.g., growth promoters). Furthermore, some methods were developed to detect rbST in bovine and buffalo serum, using liquid chromatography/tandem mass spectrometry [9,10]. However, due to different rbST variants available with varying terminal amino-acid compositions, and the low circulation levels of rbST in blood, it is difficult to detect rbST using these methods [11]. Therefore, the use of indirect methods could be an alternative for screening the use of rbST in cattle. In the past few years, transcriptomics emerged as a promising tool for evaluating the use of illicit substances in cattle [12]. Moreover, some studies used qPCR to develop a screening panel that could elucidate the use of growth promoters in cattle [13,14]. Some studies evaluated the gene-expression modifications caused by rbST in various tissues of dairy cattle [15,16,17]. However, none of these studies focused on the development of a screening panel to be used routinely in monitoring the illicit use of rbST in dairy farms.

Routine controls of rbST ab(use) should be carried out at different time points in dairy farms to avoid the market release of milk produced using rbST. Therefore, post-mortem samples from the liver or muscle are totally discarded in this type of control. From a practical point of view, only matrices that can be collected in vivo are really interesting for routine controls. The matrix which is easiest to collect in a dairy farm is that of milk, and the somatic cells present in milk are a good alternative for transcriptomic assays [18]. The collection of other matrices requires direct contact with the animal. A good example of this is blood, which is commonly used for in vivo transcriptomics assays [19]. Another option to carry out in vivo transcriptomics assays is the hair follicle, as hair is a common matrix to detect the use of illicit substances in cattle [20]. However, until now, there are no studies focusing on the use of hair follicles for screening transcriptomics assays to control the use of banned substances in cattle.

Therefore, the aim of this study was to compare the potential of three matrices (milk somatic cells, blood, and hair follicles) to detect the ab(use) of rbST in dairy cattle, using gene expression. A total of nine dairy cows were involved in the study (six rbST-treated cows and three control cows). A total of 15 target and three reference genes were selected, and their expressions in three different matrices were determined using a high-throughput real-time platform, allowing the simultaneous analysis of various genes in a wide range of samples.

## 2. Results

### 2.1. RNA Isolation and Quality

The amount of RNA isolated from the samples varied across matrices (Table 1). The amount of RNA isolated from somatic cells was significantly more abundant (*p* < 0.001) than that in blood and hair follicles. Furthermore, the ratio of absorbances at 260 and 280 nm (A260/A280) was significantly different (*p* < 0.001) across matrices. The RNA extracted from hair follicles presented the highest A260/A280 ratio, with a mean value of 1.892, while the RNA from blood presented a mean A260/A280 ratio of 1.809, and the RNA from milk somatic cells presented the lowest A260/A280 ratio, with a mean value of 1.710. The RNA integrity number (RIN) values showed a similar tendency. The RIN values of RNA extracted from hair follicles and blood were significantly higher than that of RNA obtained from milk somatic cells.

### 2.2. Reference Genes

Three different genes, namely ubiquitously expressed prefoldin-like chaperone (*UXT*), ribosomal protein S9 (*RPS9*), and mitochondrial glycerol 3-phosphate acyltransferase (*GPAM*), were evaluated as internal controls for this gene-expression study. Their stability was evaluated using the Bestkeeper^®^ software. With this software, the standard deviation (SD) of crossing-point values (CPs) of candidate reference genes was calculated, as well as Bestkeeper indexes using the SD values of candidate genes. The most stable genes exhibit the lowest variation, and any studied gene with an SD higher than 1 can be considered inconsistent.

There were differences in the expression of reference genes. The expression of all three reference genes included in this study was detected in somatic-cell samples. However, in blood and hair-follicle samples, only the expressions of *UXT* and *RPS9* were detected. Therefore, the stability of the three genes was calculated in somatic cells, while only the stability of *UXT* and *RPS9* was calculated in blood and follicle samples. Table 2 shows the SD (±CP) of the selected genes from each type of matrix.

The *GPAM* gene was only expressed in somatic cells; in the samples analyzed for this study, the SD (±CP) was higher than 1, and could be considered as inconsistent. The SD (±CP) of the other two genes was lower than 1, and therefore, could be considered as consistent. Furthermore, in hair follicles, the SD (±CP) was similar for both genes (*UXT* and *RPS9*), while in the blood and in somatic cells, *RPS9* showed lower SD (±CP) values. These results indicate a better stability of this gene in comparison with *UXT*. The blood samples presented the best Bestkeeper index. In the case of somatic cells, the use of only *UXT* and *RPS9* allowed us to obtain a better Bestkeeper index. Hair follicles presented the worst Bestkeeper index. In this study, only the *UXT* and *RPS9* genes were used to normalize the expression of target genes.

### 2.3. Expression Pattern of Target Genes Analyzed

**Figure 1:**
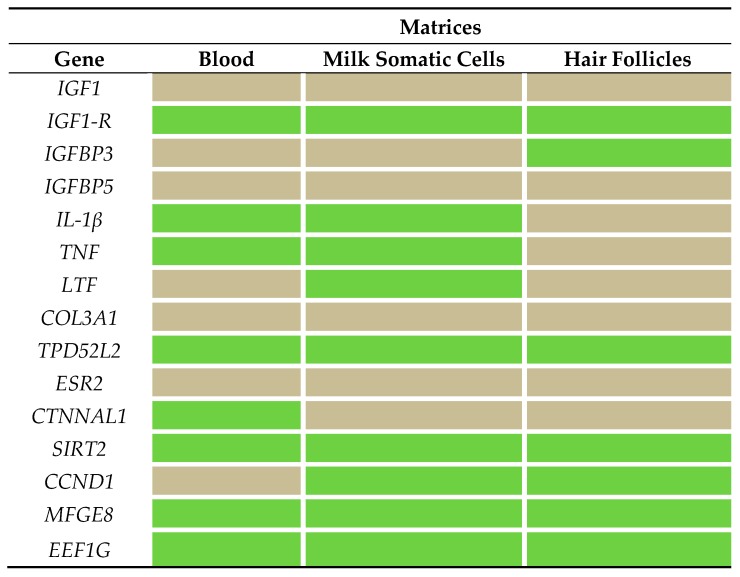
Gene-expression patterns in blood, milk somatic cells, and hair-follicle samples. Green cells represent expression of the target gene, and gray cells represent no expression of the target gene, in the samples of each matrix. For a list of the gene names evaluated, see Table 3.

The expressions of three genes, namely insulin-like growth factor binding protein 5 (*IGFBP5*), collagen type III alpha 1 chain (*COL3A1*), and estrogen receptor 2 (*ESR2*), were not detected in the matrices tested (Figure 1). In addition, the expression of *IGF-1* was only detected in one cow in the milk somatic cells. The number of target genes in which expression was detected in this study varied in each matrix. In milk somatic-cell samples, the expressions of nine genes were detected, and the transcription of lactotransferrin (*LTF*) was only detected in this matrix. In blood samples, the expressions of eight genes were observed, and the transcription of catenin alpha-like 1 (*CTNNAL1*) was only detected in this matrix. Finally, in hair-follicle samples, the transcriptions of seven genes were observed, and the expression of insulin-like growth factor binding protein 3 (*IGFBP3*) was detected in this matrix.

#### 2.3.1. Expression of Target Genes in Blood Samples

**Figure 2:**
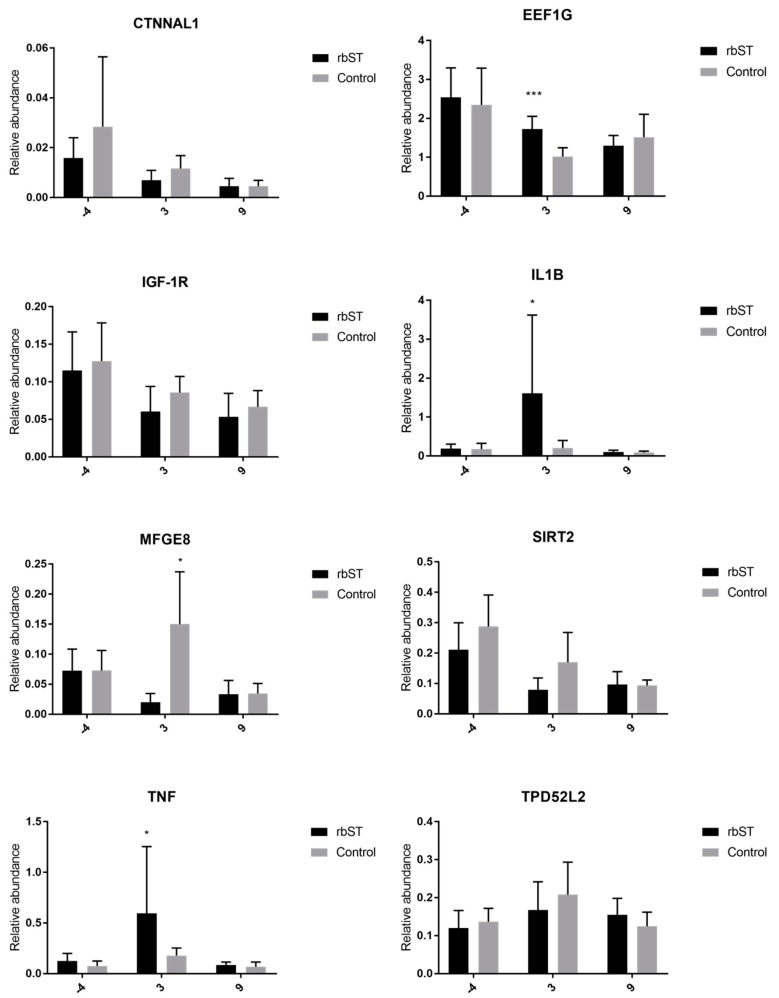
Relative abundance of target genes expressed in blood samples before the administration of recombinant bovine somatotropin (rbST), and on the third and ninth days after rbST administration. The bars represent the mean value of each group (rbST and control). The value used for each animal is the mean of three replicates; * *p <* 0.05, *** *p <* 0.001.

Gene-expression assays were carried out the week before the first rbST administration, and on the third and ninth days after rbST administration. Genes differently expressed between rbST and control samples were only detected on the third day after rbST administration (Figure 2). Specifically, eukaryotic translation elongation factor 1 gamma (*EEF1G*), tumor necrosis factor (*TNF*), and interleukin 1 beta (*IL-1β*) were significantly upregulated in rbST samples. However, milk fat globule epidermal growth factor (EGF) 8 (*MFGE8*) was significantly downregulated in the rbST group on the third day.

#### 2.3.2. Expression of Target Genes in Milk Somatic-Cell Samples

**Figure 3:**
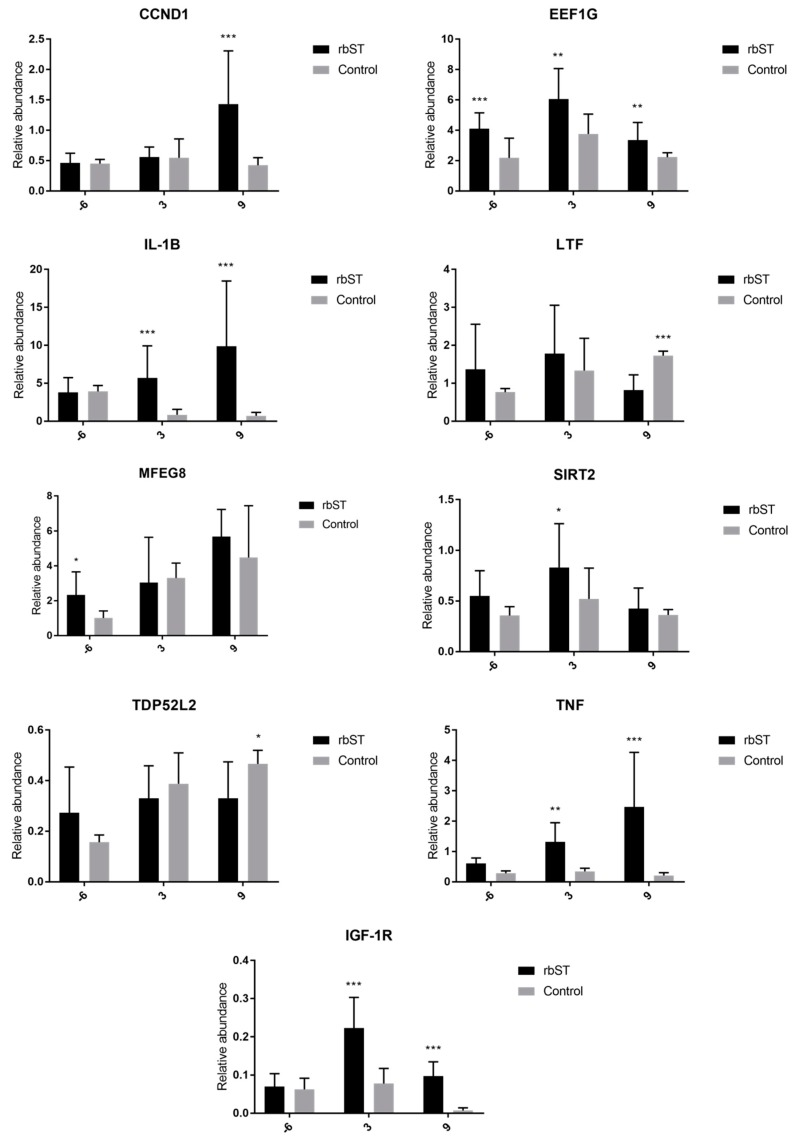
Relative abundance of target genes expressed in milk somatic-cell samples before rbST administration, and on the third and ninth days after rbST administration. The bars represent the mean value of each group (rbST and control). The value used for each animal is the mean of three replicates. * p < 0.05, ** p < 0.01, *** p < 0.001.

The *IL-1β*, insulin-like growth factor 1 receptor (*IGF-1R*), and *TNF* genes were significantly upregulated (Figure 3) in the rbST group on the third and ninth days after recombinant-hormone administration. The cyclin D1 (*CCND1*) gene was significantly upregulated in the rbST group on the ninth day. On the other hand, *LTF* and tumor protein D52-like 2 (*TDP52L2*) were significantly upregulated on the ninth day in the control group. The transcription of *EEF1G* was significantly different between both groups for all sample points, including those before rbST administration, and *MFEG8* expression was higher in the rbST group on the sixth day prior to administration, but not after rbST administration.

#### 2.3.3. Expression of Target Genes in Hair-Follicle Samples

**Figure 4:**
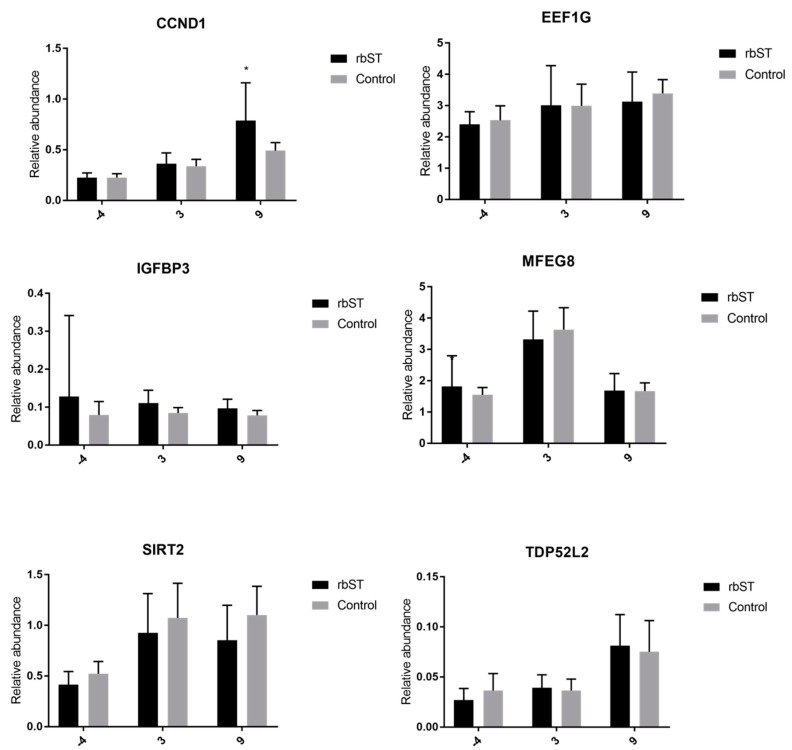

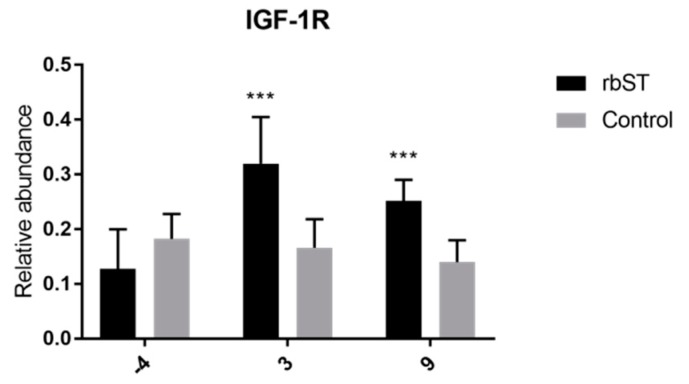
Relative abundance of target genes expressed in hair-follicle samples before rbST administration, and on the third and ninth days after rbST administration. The bars represent the mean value of each group (rbST and control). The value used for each animal is the mean of three replicates. * p < 0.05, *** p < 0.001.

In this matrix, significant differences between the rbST and control groups were only detected in the expression of *IGF-1R* on the third and ninth days, and also in *CCND1* on the ninth day (Figure 4). The other genes showed no significant differences for all time points analyzed.

## 3. Discussion

On the basis of the results observed in this study, milk somatic cells may be highlighted as the best candidate for a target matrix in rbST gene-expression analysis, as it showed the highest differences in the selected genes. Transcription patterns are expected to be tissue-specific, and, on this basis, a target matrix should be selected following action-based criteria. The mammary gland is considered one of the principal target tissues of rbST, and as such, it provided the most interesting transcription pattern. Additionally, it is worth mentioning that the stability of the selected reference genes varied according to the type of matrix. Hence, it is relevant to explore new reference candidates for hair follicles, as this matrix showed the poorest stability for the housekeepers selected in this study. These results highlight the importance of evaluating various candidates to find those with better stability, as reference genes are one of the most important aspects of RT-qPCR assays [21].

In this study, various target genes were included with the aim of finding a biomarker signature of rbST administration. Samples of the three matrices evaluated were collected at three different time points (before rbST administration, and on the third and ninth days after rbST administration). The expression of insulin-like growth factor 1 (*IGF1*) was evaluated in this study, and its expression was only detected in one cow in the somatic cells. For the remainder of the animals, its expression was not detected in any of the evaluated matrices. It is possible that the transcription of *IGF1* associated with rbST administration is focused in the liver, and *IGF1* synthetized in this organ circulates to the other tissues, such as the mammary gland, where rbST exerts an indirect function through an IGF1 molecule [22]. Furthermore, the expression of *IGF-1R* (the receptor of IGF1) was detected in all three matrices. The expression of *IGF-1R* was significantly upregulated, both in somatic cells and hair follicles, in the rbST group in comparison with the control group on the third and ninth days. However, in blood, there were no differences across groups. Accordingly, Castigliego et al. [15] observed no influence of rbST on the expression of *IGF-1R* in the muscle of treated cows. These data highlight the tissue-specific effect of rbST, and the need for performing the selection of candidate target genes according to the matrix that is going to be analyzed. Moreover, the expression of genes coding for IGF-1-binding proteins was analyzed, and only the expression of *IGFBP3* was detected in hair follicles; however, no significant differences were observed across time points. These results discredit the use of the expression of genes coding for IGF-1-binding proteins as biomarkers of rbST treatment in the matrices studied.

Two genes related to the immune system (*IL-1β* and *TNF*) were included in this study. The expression of these genes was detected in milk somatic cells and in blood, but not in hair follicles. The expressions of *IL-1β* and *TNF* were clearly upregulated in milk somatic cells on the third and ninth days after rbST administration. In blood, the differences across groups for these genes were only significant on the third day, and these differences were less significant than in milk somatic cells. A meta-analysis study showed that rbST administration increases health problems in dairy cows [5]. This exogenous substance can alter the immune system of cows, and this could explain the upregulation of the immune-system-related genes, *TNF* and *IL-1β*, in the treated group. Also, in previous screening transcriptomics studies, it was observed that anabolic treatments caused an upregulation of *IL-1β* in blood and vaginal smear cells [13,23]. In addition, other processes involving infections such as subclinical mastitis can increase the expressions of *IL-1β* and *TNF* in mammary glands [24,25]. Therefore, these genes cannot individually be considered as specific markers of rbST administration. Therefore, in screening transcriptomic studies, it is necessary to include a range of genes, as one gene can be up- or downregulated on the basis of several factors. As the number of genes included in the panel increases, so does the discrimination power of the designed panel.

Other genes with functions involving cell cycle, proliferation, differentiation, and adhesion were included in this study. It is known that rbST increases milk synthesis by increasing the turnover (proliferation/apoptosis) and activity of mammary epithelial cells, indicating that rbST strongly influences metabolic pathways that regulate cell turnover/cycle and metabolism [25]. Accordingly, the *CCND1* gene plays an important role in cell physiopathology because its dysregulation is strongly related to a drive in inappropriate cell division, and the generation of genome instability, generating neoplastic growth [26]. This gene was significantly upregulated on the ninth day in the rbST group for milk somatic cells and hair follicles. These results highlight the close relationship between rbST and cell-cycle regulation. The administration of this exogenous substance causes an overexpression of *CCND1* that can result in the activation of cells in the G_0_ phase. Therefore, the *CCND1* gene is a promising marker for detecting the use of rbST in dairy cattle, using milk somatic cells and hair follicles. However, in blood, the expression of *CCND1* was not detected. On the other hand, the *EEF1G* and *MFGE8* genes were significantly upregulated and downregulated, respectively, in the rbST group on the third day for blood samples. A previous study in the mammary tissue of rbST-treated cows also observed an upregulation of *EEF1G* six days after hormone administration [16]. *EEF1G* is involved in translation elongation via the transport of aminoacyl transfer RNAs (tRNAs) to the ribosome for protein synthesis [27]. Therefore, the upregulation of this gene is associated with a higher level of protein synthesis, potentially activated by the exogenous administration of rbST. However, in milk somatic cells and hair follicles, it was not possible to establish a relationship between the expression of this gene and rbST treatment. The *MFGE8* gene is an essential factor for attenuating inflammation and inhibiting inflammasome-induced IL-1β production [28]. As such, it is remarkable that, while *TNF* and *IL-1β* expressions were upregulated in blood samples, *MFGE8* was downregulated in treated cows on the third day. These results highlight the inverse relationship between the expressions of these genes. Due to its role in attenuating inflammation, *MFGE8* could be downregulated as an inflammatory response to the external administration of rbST.

In the case of sirtuin 2 (*SIRT2*), this gene was upregulated on the third day in the rbST group for milk somatic cells, and no differences were observed in the other two matrices. *SIRT2* is closely related to cell activity, and it is involved in the cell cycle. *SIRT2* is required for normal mitotic progression and the prevention of chromosomal instability. *SIRT2* levels are greatly increased during mitosis, and its inhibition interferes with cell-cycle progression [29]. This result again highlights the influence of exogenous rbST on cell-cycle regulation. Finally, *TDP52L2* and *LTF* were downregulated in the rbST group on the ninth day, and no rbST influence or expression was detected in the other two matrices tested. These data highlight the influence of matrices in the differences observed between two groups using gene expression, and the importance of selecting genes for the screening panel according the matrix that is analyzed.

The transcriptional modifications after rbST administration were influenced by both time and the matrix used. Blood and milk somatic cells are good candidates to be used as routine matrices for screening purposes. In the case of hair follicles, *IGF-1R* and *CCND1* were upregulated after rbST administration. However, two genes alone are seemingly not enough to detect rbST administration. However, the simultaneous analysis of multiple matrices can be combined. Based on the results observed across the different matrices, *IL-1β*, *TNF*, *IGF-1R*, and *CCND1* are good candidates to be included in a screening panel. Although the transcription of *EEF1G* and *MFGE8* was influenced by rbST in blood samples, they were upregulated in milk somatic cells before hormone administration, and their use in a panel should only be considered in blood matrices. It is important to note that, in the presented study, control animals were not injected with excipients of Lactotropina^®^, as its exact composition is not declared by the manufacturer. On the other hand, in real-farm conditions, control animals are not treated with excipients. Nonetheless, it could be interesting to prepare excipient injections of a known composition for control animals in any future research focused on rbST analysis.

OpenArray^®^ technology allows the simultaneous analysis of various genes in a wide number of samples [30]. Therefore, in routine controls, three matrices can be collected from the same farm and can be analyzed simultaneously. The overexpression of various selected genes in different matrices can be a good indication of rbST administration. Realistically, it is not possible to know when rbST was administered in a farm. Ideally, every day during lactation should be controlled. However, from a practical point of view, this suggestion is unrealistic. Therefore, a random collection of samples (for instance, once per week and on alternating week days) is proposed. As rbST is injected on a regular basis throughout lactation (bi-weekly) in all lactating cows on the farm, rbST administration should be detected at some point using an optimal sampling plan. The gene-expression data obtained from the samples collected have to be compared with a gene-expression dataset from a control population. The samples presenting higher values of gene expression proposed in this paper should be considered as indicative of potential rbST administration. Finally, the gene-expression method described here is hereafter proposed as a screening method. The illicit use of rbST can be confirmed using other methods. It is also important to highlight the high-throughput capacity of this real-time PCR approach, making the sampling plan a feasible one.

## 4. Materials and Methods

### 4.1. Animals and Treatments

Nine Holstein cows of first and second lactation stages with an age ranging from 1.5–4 years were chosen from a herd of cows, and were housed separately at the same farm. Cows were fed twice a day with ad libitum access to fresh water. The cows were divided into two groups: the control group, composed of three cows, and the treated group, composed of six cows. The treated group, with an average of 67 ± 4 days in lactation, was subcutaneously administrated with 500 mg of rBST (Lactotropina^®^, Elanco^®^, Eli Lilly, Mexico D.F, Mexico), according to the manufacturer’s recommendations.

Experimental procedures were performed after evaluation and upon approval of the corresponding regional authorities (Service of Livestock Farming of the Provincial Government of Lugo, Regional Ministry of Rural Affairs, Galicia), in accordance with EU guidelines and national laws on animal experiments, in particular, Directive 2010/63/EU on the protection of animals used for scientific purposes, and its transposition into national law. All procedures were performed respecting animal welfare and causing no further pain, suffering, distress, or lasting harm than the equivalent of that caused by the introduction of a needle in accordance with good veterinary practice.

### 4.2. Matrices Collection

#### 4.2.1. Milk Somatic-Cell Collection

**Figure 5:**
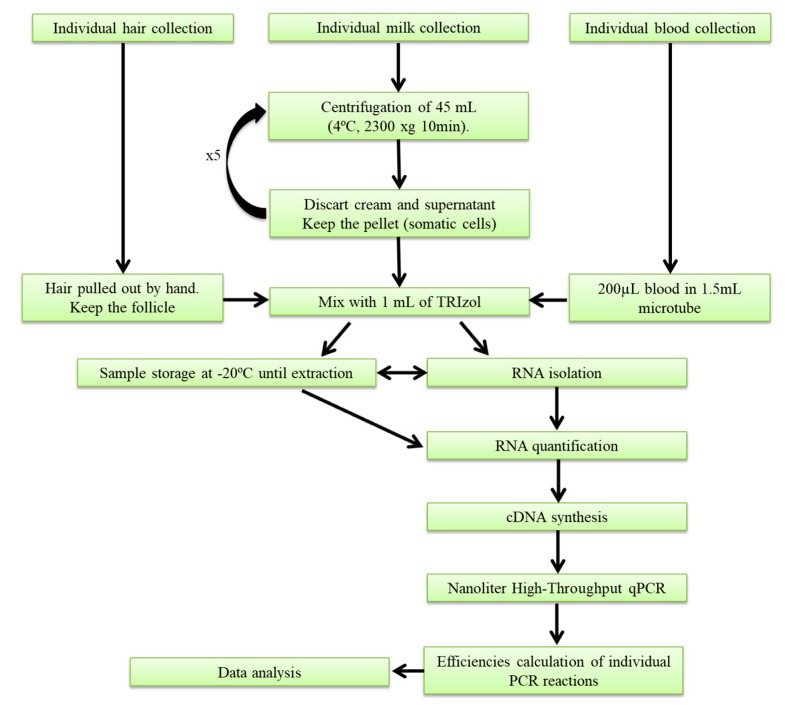
Workflow of matrix collection and gene-expression analysis.

Milk somatic-cell collection was carried out before rbST treatment, and on the third and ninth days after rbST administration. Briefly, two liters of homogenized milk from the morning milking of each cow was collected at the milking parlor in sterile bottles (Deltalab, Barcelona, Spain), and was immediately transported in refrigerated conditions to the laboratory. Before milking, the udder was cleaned, and the initial contaminated milk streams were dismissed. The remaining milk that was not collected for experimental assays was discarded. A total volume of 225 mL of fresh milk was used to collect the milk somatic cells (MSCs) for analysis. The protocol followed for isolating MSCs is shown in Figure 5. Briefly, 50-mL conical centrifuge polypropylene tubes were filled with 45 mL of milk, and were centrifuged at 4500 rpm for 10 min at 4 °C. The supernatant (fat and whey) was discarded, keeping the pellet, and then, the tubes were again filled with 45 mL of milk, and centrifuged once more. This step was repeated five times for each sample in the same conical plastic tube in an effort to concentrate the pellet. Then, the milk pellet containing the MSCs was mixed with 1 mL of TRIzol (Ambion^TM^, Thermo Fisher Scientific, Waltham, MA, USA), before being transferred to a 1.5-mL microtube and stored at −20 °C until further use. Additionally, a total of three MSCs samples from other dairy farms were collected following the same procedure.

#### 4.2.2. Blood Collection

Blood samples were taken at three different time points from each animal involved in the experiment. Pre-dose samples were taken prior to treatment. Further samples were taken on the third and ninth days after rbST administration. A volume of 3 mL of blood was extracted from the tail using tubes with tripotassium ethylenediaminetetraacetic acid (K_3_EDTA) (Vacuette^®^, Greiner bio-one, Madrid, Spain), and the samples were transported to the laboratory in refrigerated conditions. Then, a total of 200 µL of whole blood was mixed with 1 mL of TRIzol reagent (AmbionTM, Thermo Fisher Scientific, Waltham, MA, USA), and the samples were frozen at −20 °C until use.

#### 4.2.3. Hair-Follicle Collection

Hair samples were collected from control and treated animals before rbST administration, and on the third and ninth days. Before hair collection, the surface of the cow chosen for sampling was cleaned with 96 °C alcohol to eliminate macromolecular contamination. The hair was pulled out by hand, and was then cut resulting in 5 mm of follicle, before being transferred to 1.5-mL microtubes. Then, the hair follicles were mixed with 1 mL of TRIzol reagent (AmbionTM, Thermo Fisher Scientific, Waltham, MA, USA), and samples were frozen at −20 °C until use.

### 4.3. Total RNA Extraction and Reverse Transcription

Total RNA was extracted using TRIzol reagent (AmbionTM, Thermo Fisher Scientific, Waltham, MA, USA). Quantification of RNA was carried out with a Qubit^®^ RNA BR Assay Kit and a Qubit^®^ fluorometer (InvitrogenTM, Thermo Fisher Scientific). The A260/A280 ratio of RNA samples was determined using a BioDrop μLITE (BioDrop, Cambridge, UK). RIN values were determined using a 2100 BioAnalyzer (Agilent Technologies, Santa Clara, CA, USA). A total of 2 µg of RNA was reverse-transcribed to complementary DNA (cDNA) using a High-Capacity cDNA Reverse Transcription Kit with RNase Inhibitor (Applied BiosystemsTM, Thermo Fisher Scientific) according to the manufacturer’s instructions. The cDNA samples were stored at −20 °C until further use.

### 4.4. Nanoliter High-Throughput qPCR

The expressions of 18 genes (Table 3) were evaluated using real-time PCR. Three genes were used as endogenous controls to calculate the relative expressions of the other 15 candidate genes. The selection of reference and target genes was based on results observed in previous transcriptomics studies, where the administration of rbST and anabolic agents to cattle was evaluated [13,14,15,16,17,31]. Gene-expression assays were carried out with a TaqMan^®^ OpenArray^®^ system (Applied BiosystemsTM, Thermo Fisher Scientific), involving a nanoliter high-throughput real-time PCR platform where 3072 reactions were performed simultaneously in the same OpenArray^®^ plate, and the primers and TaqMan^®^ probes were preloaded in the plates by the company. A plate design of 18 assays in triplicate, and 56 samples was chosen. Real-time PCR reactions were performed according to the TaqMan^®^ OpenArray^®^ protocol. Briefly, in a 384-well plate, 1.2 µL of each cDNA sample was mixed with 3.8 µL of TaqMan^®^ OpenArray^®^ Real-Time PCR Master Mix (Applied BiosystemsTM, Thermo Fisher Scientific). The PCR reaction mixtures were loaded automatically into the OpenArray^®^ plates using an OpenArray^®^ AccuFill™ System (Applied BiosystemsTM, Thermo Fisher Scientific). The following real-time PCR protocol was used: 40 cycles at 95 °C for 15 s, and 60 °C for 1 min.

### 4.5. RT-PCR Data Analysis

The LinRegPCR software (version 2017.0, J.M Ruijter, Amsterdam, The Netherlands) was used to analyze the raw real-time PCR data [32,33]. LinRegPCR imports non-baseline-corrected data, and performs a baseline correction on each sample. Then, a window of linearity is determined, and linear regression analysis is used to determine the PCR efficiency per sample from the slope of the regression line. The mean PCR efficiency of each amplicon tested and the Cq value per sample were used to calculate a starting concentration (N_0_) per sample, expressed in arbitrary fluorescence units. After that, the Factor Correction qPCR software was used to remove multiplicative between-session variation in experiments [34]. A session factor is used to correct the observed data, and it can be calculated from a matrix of between-session ratios, or estimated using a maximum-likelihood approach. Corrected values are obtained by dividing the observed values by the session factor. Finally, the gene-expression ratio was calculated by dividing the N_0_ of the target gene by the N_0_ of the geometric mean of the three reference genes. The *UXT*, *RPS9*, and *GPAM* genes were used as reference genes, and they were validated using the BestKeeper^®^ tool for the determination of stable housekeeping genes [21].

### 4.6. Statistical Analysis

A one-way ANOVA and a Tukey’s honest significance test were used to determine the differences across matrices in RNA concentration, A260/A280 ratio, and RIN values. A Kolmogorov–Smirnov test was used to evaluate data normality. These statistical analyses were performed using the IBM SPSS Statistics software for Windows (SPSS Inc., Chicago, IL, USA). For comparison of transcriptomics results between the control and rbST groups at different time points, an unpaired *t*-test was used when data were normally distributed, and a Mann–Whitney test was used when data were not normally distributed. These statistical analyses were performed using the GraphPad Prism 7 software for Windows (La Jolla, San Diego, CA, USA). 

## 5. Conclusions

The three matrices selected in this study were shown to be promising candidates for use in cattle gene-expression studies. The RNA quality was matrix-dependent, and the collection and isolation of RNA should be carried out carefully to achieve better RIN and A260/A280 ratio values. The *CCND1*, *IL-1β*, *TNF*, and *IGF-1R* genes were shown to be potential biomarkers of rbST treatment. Future studies should focus on searching for new genes that could be included in the transcriptomic panel. Also, they should carry out analysis of a wide range of non-rbST-treated animals, with the aim of establishing a gene-expression baseline of selected genes which can be compared with samples collected in routine controls.

## Figures and Tables

**Table 1:** The concentration of RNA, the ratio of absorbances at 260 and 280 nm (A260/A280), and the RNA integrity number (RIN) values of RNA isolated from the three matrices.

Matrix	RNA Concentration (ng/µL)	A260/A280 Ratio	RIN Value
Hair follicles	53.30 ± 11.97 ^b^	1.89 ± 0.06 ^a^	7.76 ± 0.41 ^a^
Blood	59.00 ± 28.02 ^b^	1.81 ± 0.04 ^b^	7.79 ± 0.40 ^a^
Somatic cells	165.42 ± 71.23 ^a^	1.71 ± 0.08 ^c^	6.10 ± 0.57 ^b^

Letters (a–c) in each column reflect significant differences.

**Table 2:** Standard deviation of crossing-point (CP) values obtained for the three reference candidates—ubiquitously expressed prefoldin-like chaperone (*UXT*), ribosomal protein S9 (*RPS9*), and mitochondrial glycerol 3-phosphate acyltransferase (*GPAM*). For hair follicles and blood, the amplification of *GPAM* was not detected. Bestkeeper indexes were calculated based on the CP values of reference genes. For somatic cells, the Bestkeeper index was calculated based on UXT-RPS9.

Matrix	*UXT*	*RPS9*	*GPAM*	Bestkeeper Index	Bestkeeper Index (*UXT*-*RPS9*)
Hair follicles	0.90	0.91	-	0.96	0.96
Blood	0.89	0.75	-	0.71	0.71
Somatic cells	0.85	0.79	1.19	0.94	0.89

**Table 3:** Genes included in this study for gene-expression assays.

Gene Symbol	Gene Name	NCBI Accession Number	Assay ID
IGF1	Insulin-like growth factor 1	NM_001077828.1	bt03252281_m1
IGF1-R	Insulin-like growth factor 1 receptor	NM_001244612.1	bt03649217_m1
IGFBP3	Insulin-like growth factor binding protein 3	NM_174556.1	bt03223809_m1
IGFBP5	Insulin-like growth factor binding protein 5	NM_001105327.2	bt03258785-g1
IL-1β	Interleukin 1 beta	NM_174093.1	bt03212745_m1
TNF	Tumor necrosis factor	NM_173966.3	bt03259156_m1
LTF	Lactotransferrin	NM_180998.2	bt03217382_m1
COL3A1	Collagen type III alpha 1 chain	NM_001076831.1	bt03249914_m1
TPD52L2	Tumor protein D52-like 2	NM_001034615.2	bt03227133_m1
ESR2	Estrogen receptor 2	NM_174051.3	bt03259198_m1
CTNNAL1	Catenin alpha-like 1	NM_001191534.1	bt04308229_m1
SIRT2	Sirtuin 2	NM_001113531.1	bt03258971_m1
CCND1	Cyclin D1	NM_001046273.2	bt03235030_m1
MFGE8	Milk fat globule epidermal growth factor (EGF) 8 protein	NM_176610.1	bt03216856_m1
EEF1G	Eukaryotic translation elongation factor 1 gamma	NM_001040487.2	bt03229629_g1
Reference genes			
UXT	Ubiquitously expressed prefoldin-like chaperone	NM_001037471.2	bt03229278_m1
RPS9	Ribosomal protein S9	NM_001101152.2	bt03272016_m1
GPAM	Glycerol 3-phosphate acyltransferase (mitochondrial)	NM_001012282.1	bt03210379_m1
